# Evolving the Diagnosis of Multiple Sclerosis: A New Landscape in Light of the 2024 McDonald Criteria

**DOI:** 10.3390/biomedicines13112590

**Published:** 2025-10-23

**Authors:** Amjad Samara, Daniel Ontaneda

**Affiliations:** Cleveland Clinic Mellen Center for Multiple Sclerosis, 9500 Euclid Ave, U10 Mellen Center, Cleveland, OH 44195, USA; samaraa2@ccf.org

**Keywords:** 2024 McDonald criteria, central vein sign, paramagnetic rim lesion

## Abstract

Timely and accurate diagnosis of multiple sclerosis (MS) allows for prompt treatment initiation that can alter the disease course and prevent disability accumulation. The 2024 revisions of the McDonald criteria aim to achieve earlier and more precise MS diagnosis by including new neuroimaging and biomarker advances. This review highlights key updates, including revised definitions of dissemination in space and time, along with new MRI features, including the central vein sign and paramagnetic rim lesions. We also describe the role of cerebrospinal fluid biomarkers, including oligoclonal bands and kappa-free light chains. The updated criteria formally incorporate incidental imaging and nonspecific symptom presentations to enable diagnosis before either a first clinical attack or progression of neurological disability. Finally, we explore emerging and promising investigational tools for future incorporation into MS diagnosis, including advanced MRI techniques, fluid biomarkers, and applications of artificial intelligence.

## 1. Introduction

Multiple Sclerosis (MS) is a chronic, immune-mediated, and neurodegenerative disease of the central nervous system (CNS) characterized by inflammation, demyelination, and neuronal loss [1]. In clinical settings, a timely and accurate diagnosis of MS is sometimes challenging due to the heterogeneity in initial presentation and the unpredictable course of the disease [2]. Early initiation of high-efficacy disease-modifying therapies (DMTs) in patients with MS has been shown to significantly change the disease trajectory by reducing new lesion formation, reducing relapse rates, slowing disability progression, and improving long-term outcomes [3]. Conversely, misdiagnosis of MS can lead to unnecessary and potentially harmful treatments, significant psychological distress for patients, and delays in providing appropriate therapy for the true underlying condition.

Over the years, the diagnostic criteria for MS have undergone multiple revisions since early versions, including the Schumacher criteria in 1965 [4]. The Schumacher criteria were mainly clinical and relied heavily on signs and symptoms of neurologic dysfunction. Later, the Poser criteria in 1983 added supportive laboratory tests, including cerebrospinal fluid (CSF) studies and visual evoked potentials [5]. The McDonald criteria were first introduced in 2001 and transformed MS diagnosis by incorporating magnetic resonance imaging (MRI) findings, enabling earlier and faster diagnosis [6]. Subsequent updates in 2005, 2010, and 2017 further refined these guidelines, aiming to enhance sensitivity [7,8,9]. The 2017 McDonald criteria shortened the time to diagnosis and likely reduced the time to reaching disability milestones [10]. However, this increase in sensitivity resulted in the unintended consequence of an increase in misdiagnosis due to incorrect application of relaxed MRI criteria.

In this review article, we aim to provide an overview of the 2024 revisions to the McDonald Criteria. We will specifically focus on how these revisions address the main areas related to integrating radiologically isolated syndrome (RIS) into the diagnostic framework, the challenges posed by symptoms not specific to MS, and MS presentations in pediatric and older populations. We will also highlight emerging trends in MS diagnosis and potential future directions.

## 2. Key Updates Related to the 2024 McDonald Criteria and the Inclusion of New Biomarkers

The McDonald diagnostic criteria have been refined over the years, aiming for early and accurate diagnosis. MRI has been used in all prior iterations of the McDonald criteria to aid in demonstrating lesion dissemination in space (DIS) and time (DIT). In the past decade, innovative susceptibility-based sequences allowed studies demonstrating the identification of pathologically specific radiological signs [11]. The central vein sign (CVS) and paramagnetic rim lesions (PRLs) are recently described imaging markers that offer greater specificity for MS pathology [12,13]. The CVS demonstrates the presence of perivenular demyelination, which is a pathological hallmark of MS [14,15]. The PRL, conversely, is an imaging biomarker of chronic active lesions [16,17]. By including these new imaging features, the 2024 revisions offer more biologically based criteria, especially useful to differentiate MS from mimics [18].

**Key Changes in the 2024 McDonald Criteria**: The 2024 revisions introduce several modifications to the established framework, mainly modifying the criteria for demonstration of DIS and DIT. These changes aim to utilize specific MRI and CSF biomarkers more prominently and move away from the need for dissemination in time. The incorporation of these biomarkers is based on data demonstrating these markers were closely associated with MS and predict a second clinical event or the development of new lesions. The main changes in the 2024 McDonald criteria include those indicated in Table 1. Notably, DIT is not strictly required under the new diagnostic criteria. Additionally, DIS with either DIT or positive CSF fulfills diagnosis of MS, as per the prior 2017 criteria.

**Optic nerve as a fifth anatomical location**: In previous versions of the McDonald criteria, DIS required lesions in at least two of four CNS anatomical locations: periventricular, juxtacortical/cortical, infratentorial, and spinal cord. The 2024 criteria expand this to include the optic nerve as a fifth anatomical location. Optic neuritis is, in fact, the initial presentation of MS in about 25% to 30% of cases [19,20]. Several prior studies have shown that inclusion of the optic nerve in the MS diagnostic criteria improves the sensitivity of MS diagnosis without compromising specificity [21,22]. Optic nerve involvement can be confirmed with orbital MRI, optical coherence tomography (OCT), or visual evoked potentials (VEPs), with no better explanation than MS for the changes identified (explicitly requiring rigorous quality control). For OCT, optic nerve involvement is defined by an inter-eye difference in the peripapillary retinal nerve fiber layer (pRNFL) of ≥6 µm or an inter-eye difference in macular ganglion cell inner plexiform layer (GCIPL) thickness of ≥4 µm. It is important to note that OCT needs to be acquired, adhering to the OSCAR-IB criteria [23]. An abnormal VEP demonstrating delayed unilateral latency of P100 or inter-eye latencies (2.5–3 SD) is used to provide evidence of optic nerve involvement. For VEP, the results need to be performed considering methodological factors specific to the center and device. Importantly, the use of OCT/VEPs as supportive evidence is contingent upon quality control, instead of isolated definitions disconnected from the clinical circumstances.

**Revised dissemination in time requirement:** The 2024 criteria update the requirements for diagnosis by moving away from the strict necessity to establish DIT. In those with typical attacks or progression from onset, if four or five anatomic locations are involved, no additional features, including DIT, are needed to diagnose MS. The 2024 criteria now allow a diagnosis with only 1 anatomical location, requiring both the presence of DIT (on MRI or positive CSF) in addition to either CVS or PRL (as below) to confirm MS.

**Figure 1:**
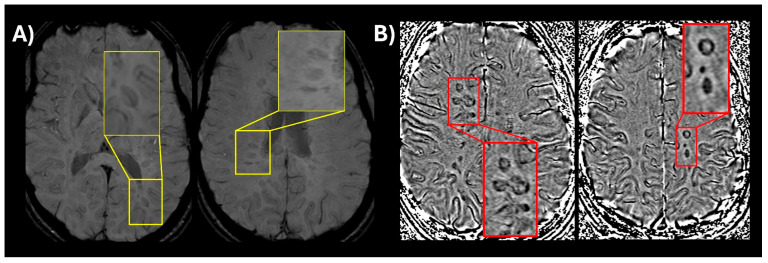
(**A**) An optimized version of susceptibility-weighted imaging (SWI) (low flip angle) in a patient with relapsing-remitting MS showing a central vein visible in multiple lesions (magnified window showing a thin dark line in the center of the lesion); (**B**) SWI phase image showing multiple paramagnetic rim lesions (PRLs) which appear as dark ring-like signal around the lesion.

**Inclusion of new imaging biomarkers for DIS (CVS and PRLs):** CVS, visualized on susceptibility-weighted imaging (SWI) or T2* segmented echo-planar imaging (T2* EPI), reflects the perivenular inflammatory pathology characteristic of MS lesions [14,24]. The presence of CVS can reliably differentiate MS lesions from mimics with high diagnostic accuracy for MS [25,26,27]. Select 6 is considered positive when six or more white matter lesions are CVS positive; or if fewer than ten white matter lesions are detected on MRI, the number of CVS positive lesions should be the majority [27]. When 2 or more anatomical locations are present, Select 6 is used to confirm MS without further requirements. Likewise, PRLs are chronic active lesions, appearing as non-enhancing T2-weighted/FLAIR lesions with a hypointense rim on phase images [16]. While PRLs are not particularly sensitive in prior retrospective studies (~55%), they demonstrate extremely high specificity (>95%) for MS [28]. A recent study conducted by the North American Imaging in MS Cooperative (NAIMS) did find that at time of presentation to a neurological center for a diagnosis of MS 86% of participants had at least 1 PRL with a specificity of 90% [29]. In summary, the new criteria incorporate CVS and PRLs in the diagnosis of MS as follows: Select 6 or ≥1 PRL lesions are sufficient for MS diagnosis, when 1 anatomical location is present in addition to DIT on MRI or positive CSF. Due to logistical and technical considerations, these two MRI features are not mandatory for MS diagnosis and represent high-specificity supportive evidence in early disease course. An example of CVS and PRLs is shown in Figure 1.

**CSF-restricted oligoclonal bands (OCBs) and the kappa-free light chains (kFLC)**: The presence of CSF-restricted OCBs indicates intrathecal antibody production, which is a characteristic feature of MS and demonstrates the autoimmune nature of the disease [30,31]. The 2017 McDonald criteria already allowed for positive OCBs to substitute for DIT. When antibodies are produced in the CNS an excess of light chains are generated as compared to heavy chains, and these can be measured in CSF. Similar to OCBs, the presence of kFLC is a characteristic feature of MS and could be used to differentiate MS from other mimics [32]. The use of kFLC is more objective, does not require specialized reader expertise, and is less labor-intensive compared to OCBs. The utility of kFLC is acknowledged in the 2024 criteria as: the kFLC index is considered an interchangeable biomarker to OCBs to establish positive CSF, particularly in settings where OCB testing is unavailable or inconclusive [33,34]. One caveat to note is the need to use kFLC in the appropriate clinical context, given potential positive test results in other diseases with intrathecal inflammation, similar to OCB. Additionally, the IgG index is not considered a sufficient substitute in this regard.

**Considerations for the MS diagnosis in pediatric and older individuals**: The 2024 criteria provide more explicit guidance for the diagnosis of MS in people at the extremes of age, i.e., late-onset MS and pediatric MS. For individuals after age 50, where presentations not specific to MS and comorbidities are more common, additional criteria should be evaluated including: (1) at least one spinal cord lesion, (2) positive CSF (OCB or kFLC), and (3) the presence of Select 6 CVS. In pediatric MS, the criteria acknowledge the unique clinical presentations commonly observed in children. The 2024 criteria should not be applied in pediatric populations at first presentation with acute demyelinating encephalomyelitis (ADEM), and MOG antibody testing is recommended in children under 12 years old. For those 18–12, MOG antibody testing is recommended in the presence of any red flags or atypical features for MS. In pediatric-onset MS, CVS positivity in more than 50% of the lesions is supportive of a diagnosis of MS. The performance of OCT/VEP in pediatric populations is less clear and will require future studies. Similarly, PRLs are less well studied in pediatric patients with MS.

## 3. Expansion of Diagnosis: Radiologically Isolated Syndrome (RIS)

RIS refers to the presence of incidental MRI findings indicative of demyelination (in a pattern typical for MS) with no neurological symptoms [35]. Initially described by Okuda and colleagues in 2009, RIS was a research construct designed to identify individuals at risk of developing clinical manifestations of MS [36]. Evidence from recent studies suggests that RIS is part of the MS pathology spectrum, and slightly over half of RIS patients will develop definite MS within 10 years [37]. Longitudinally, several prognostic indicators have been identified for the development of clinical manifestations of MS, including male sex, younger age, spinal cord involvement, and infratentorial lesions [37,38]. OCB positivity has consistently been associated with the highest risk of progression, and more recently, elevated levels of neurofilament light chain (NfL) in CSF or serum have emerged as potential predictors of neuronal damage and subclinical disease activity [39]. The CVS has been studied extensively in RIS and has been shown to differentiate those at the highest risk of developing clinical manifestations of MS [40,41].

In all prior diagnostic criteria, MS diagnosis could not be made in the absence of typical clinical attacks or progression, and when incidental radiological findings were suggestive of the diagnosis. The 2024 McDonald Criteria revise this position by allowing MS diagnosis in patients with incidental findings suggestive of demyelination (RIS) under specific conditions. In patients with incidental findings of demyelination, a diagnosis of MS can be made in the presence of lesions in two or more anatomical locations with additional demonstration of either DIT on MRI, positive CSF, or the presence of Select 6 CVS. These recommendations are based on the evidence that OCBs and CVS increase the risk of developing clinical symptoms of MS [39,41].

Although the diagnostic criteria are not treatment criteria, the ability to diagnose MS in those with RIS will have treatment implications. The combination of clinical, radiological, and CSF testing features may help identify individuals with RIS most likely to benefit from early therapeutic intervention. Historically, a conservative approach with clinical and radiological monitoring was favored. However, recent clinical trial data challenge this paradigm; two randomized controlled trials of dimethyl fumarate and teriflunomide in individuals with RIS demonstrated a delay in the time to the first clinical event [42,43]. These findings, along with results from smaller observational cohorts, support the notion that early initiation of DMTs in RIS individuals may delay or prevent the onset of clinically definite MS. Treatment decisions must be individualized, balancing the potential benefits of early intervention against the risks and burden of long-term therapy. For lower-risk individuals or those preferring watchful waiting, continued surveillance with regular clinical assessments and serial MRI could be used [44]; however, philosophically, a biologically confirmed diagnosis of MS should prompt treatment of MS as one would in any patient carrying that diagnosis.

## 4. Expanding the Diagnostic Criteria: Symptoms That Are Not Specific to MS

Historically, the McDonald criteria were developed and validated for patients with typical MS attacks, such as optic neuritis and partial transverse myelitis or progression of neurological disability. On the other hand, presentations not specific to MS, like isolated headaches, fatigue, and nonspecific neurologic deficits, often pose diagnostic uncertainty and are common. While these symptoms may ultimately indicate MS, they frequently overlap with alternative etiologies [2]. Therefore, diagnosing MS in patients with non-specific symptoms often remains challenging or leads to misclassification under broader CNS inflammatory categories. In such cases, an accurate MS diagnosis relies on careful integration of clinical features with supportive neuroimaging and CSF findings. The 2017 revisions to the McDonald criteria aimed to address some of these issues by stressing the importance of applying the criteria within the “appropriate clinical context” and establishing that there is truly “no better explanation” for the symptoms and radiographic findings [45]. In the 2024 revisions, the diagnosis of MS can now also be made in those with symptoms not specific to MS. Philosophically, making a diagnosis in a person without symptoms (RIS) should not be any less difficult than making it in individuals who have some neurological symptoms, albeit nonspecific or not rising to the level of a typical clinical attack or progression. Therefore, in the 2024 revisions, a diagnosis of MS can be made in those with symptoms not specific for MS who have one or more lesions in two or more anatomical locations with additional demonstration of either DIT on MRI, positive CSF, or positive Select 6 CVS. On the other hand, the expansion of criteria to asymptomatic patients could raise clinical dilemmas and ethical challenges with therapeutic implications, inappropriate exposure to DMT, and the psychosocial burden of a chronic condition mislabel. Therefore, correct application of the criteria will be critical, especially when used outside of typical presentations or with over-reliance on MRI, particularly in lesions lacking characteristic features, size, or location [46]. A balance between clinical acumen and understanding of criteria limitations would also be necessary to avoid “out-of-context” test interpretations.

## 5. Reducing Misdiagnosis

The 2024 revisions of the McDonald criteria explicitly acknowledge the role of objective biomarkers to improve diagnostic accuracy, particularly in patients who are at risk of misdiagnosis. This group includes those with risk factors for WM lesions that may mimic MS (migraine headache, vascular comorbidities, and those above age 50). In older individuals, there is a higher prevalence of comorbidities like vascular disease and migraine, which increases the risk of misdiagnosis. In cases of older individuals and those with risk factors, the presence of spinal cord lesions can be used to support the diagnosis of MS. These considerations also include the presence of positive CSF testing (OCB/kFLC) and positive Select 6 CVS. The new criteria explicitly recommend these additional tests to reduce the risk of misdiagnosis in older individuals.

The formal inclusion of CVS and PRLs as supportive imaging markers enhances the specificity of MRI-based diagnosis and reduces the weight placed on purely syndromic presentations. Supportive evidence stems from studies that consistently demonstrate a lesion-level prevalence of ≥40% of CVS is highly specific to MS when compared to mimics, including migraine, small vessel ischemic disease, and other inflammatory conditions [14,47,48]. Multiple studies and a recent meta-analysis demonstrate the diagnostic performance of CVS for MS [28]. In addition, two multi-center studies demonstrate that CVS has similar or improved performance when compared to OCBs, a test already used as a specific biomarker of MS [27,49]. Similarly, PRLs correlate with tissue-based MS pathology and have been linked with clinical progression and poor repair potential, making them highly relevant in early and non-specific presentations [16]. PRL also demonstrates excellent specificity for MS in meta-analysis [28]. As noted above, an additional multi-center study by the NAIMS group recruited patients at the time of diagnostic evaluation and found both high sensitivity (86%) and specificity for the diagnosis of MS (90%) [29]. These findings suggest that susceptibility-based markers aid in distinguishing MS from mimics, and PRL may also have potential prognostic implications. Additionally, the 2024 criteria integrate the role of OCBs and kFLC, especially when clinical and imaging findings are not sufficient to make a diagnosis. When interpreted alongside specific imaging findings, these CSF markers can push diagnostic probability past the threshold necessary to establish the diagnoses and initiate DMTs confidently.

Although the revisions in the diagnostic criteria aim to improve specificity, the persistence of misdiagnosis could also reflect clinician misapplication rather than the criteria’s intrinsic validity. Structured training in the interpretation of these new diagnostic criteria and implementation of safeguards remains essential to reduce the risk of misdiagnosis. Therefore, while updated criteria represent an incremental step toward diagnostic precision, successful implementation will ultimately rely on clinician judgment and adherence to evidence-based interpretation standards.

## 6. A Unified Framework for the Diagnosis of MS

In previous diagnostic criteria, MS diagnosis was separated based on disease presentation, as relapse onset and progression onset. The disease course descriptors have helped to guide clinical care, research, and therapy approvals. However, increasing evidence supports the idea that MS should be viewed as a disease continuum influenced by various pathological processes and not as distinct entities [11]. The apparent transition to progressive disease reflects not a sudden change but a gradual increase in widespread tissue damage and decreased compensatory mechanisms, which is further impacted by aging-related vulnerability [50]. This evolving framework emphasizes the need to view MS not as separate categories but as a spectrum where overlapping pathophysiological mechanisms influence individual disease courses.

In line with this conceptual shift, recent studies have questioned the distinction between diagnostic frameworks for relapsing and progressive MS [11,51]. Studies have shown that applying unified DIS algorithms across both relapsing and progressive PPMS groups provides similar diagnostic accuracy, especially when CSF biomarkers are included [51]. Additional studies of patients with suspected progressive MS have shown that DIS criteria for relapsing MS, especially when modified to include optic nerve or multiple spinal cord lesions, perform well when combined with DIT or positive CSF testing [52]. Therefore, the 2024 revisions to the McDonald criteria diverged from the previous distinction between relapsing and primary progressive criteria and shifted toward a single, unified diagnostic approach framework. For primary progressive MS, the diagnosis still requires evidence of clinical progression over at least 12 months; however, all other elements for diagnosis are the same with the exception that two or more spine lesions are sufficient to demonstrate DIS in patients with a progressive course. The latter was considered necessary given a proportion of patients with a relative paucity of brain lesions among those labeled as primary progressive MS.

## 7. Concluding Remarks and Future Directions

Successful implementation of the 2024 McDonald Criteria will require overcoming several practical challenges. Identifying features like the CVS and PRLs demands access to specialized imaging sequences and radiological expertise, which may not be universally available. Similarly, while CSF-specific OCB remains widely used, broader adoption of kFLC will require standardized laboratory protocols and improved accessibility. To ensure consistent and accurate application of the revised criteria, educational programs for neurologists, trainees, and neuroradiologists are essential. Most importantly, despite these advancements, diagnostic accuracy still depends on solid clinical judgment to distinguish MS from its mimics, remembering that the first step in establishing an MS diagnosis is excluding alternative explanations to clinical and paraclinical features.

Technological advancements in imaging, fluid biomarkers, and computational tools will continue to shape the future directions of the diagnosis of MS. In the imaging arena, quantitative MRI, automated image analysis, and ultra-high field MRI (7T) have emerged as the next frontier. Although these technologies are currently only used in research settings, their applications will enhance the detection of microstructural tissue changes associated with MS pathology. For example, volumetric measures such as cortical thickness and thalamic volume serve as proxies for neurodegeneration and as an outcome measure in early-phase clinical trials. Future application of these measures could be complementary in improving both early diagnosis and longitudinal monitoring when combined with traditional lesion metrics [53]. Ultra-high field MRI (7T) further enhances diagnostic precision by improving spatial resolution, allowing for the detection of features with high specificity for MS, such as CVS and PRLs [54]. The detection of cortical lesions, previously limited at clinical field strength, is also significantly improved at 7T, offering particular value in early diagnosis of MS [55]. Automated tools for CVS detection, PRL detection, and volumetric analysis may put additional diagnostic tools at the fingertips of clinicians but will require a streamlined implementation and validation. Low-field MRI is another technology that may expand where and how often an MRI can be done and may have a role, particularly in underserved areas [56]. Complementing imaging advances, fluid biomarkers such as serum and NfL and Glial fibrillary acidic protein (GFAP) have gained prominence as indicators of axonal injury and astrocytic activation, respectively. Serum NfL levels correlate with acute disease activity, lesion load, and long-term brain atrophy, making it a valuable tool for both early diagnosis and monitoring subclinical disease activity [57,58]. Similarly, elevated GFAP levels are associated with disease progression independent of relapse activity and may provide complementary information to NfL [59]. Although promising, the diagnostic relevance of these biomarkers remains untested, and their lack of specificity still needs to be addressed. Nevertheless, these advancements will inevitably augment the efforts to overcome current limitations related to the application of the new diagnostic criteria.

Finally, artificial intelligence is increasingly being integrated into MS research and clinical practice, particularly in imaging [60,61]. For example, automated lesion segmentation tools now achieve high accuracy in delineating T2 lesion volume, CVS lesions, and PRL, reducing inter-rater variability and enabling scalable, objective assessments of disease burden over time [62,63]. Beyond imaging, machine learning models trained on multimodal data, including MRI, biomarkers, and clinical features, are being utilized to predict the development of MS in at-risk populations, estimate the risk of disability progression, and forecast treatment response. These developments support the broader vision of precision medicine in MS, where individualized risk modeling could inform both diagnosis and therapeutic strategies.

## Figures and Tables

**Table 1:** New imaging and CSF markers included in the 2024 McDonald Criteria.

Biomarker	Definition	Inclusion in the McDonald Criteria
Central vein sign (CVS)	A brain lesion with a central, hypointense dot or line, indicating a central vein. Visualized in ≥1 plane in appropriate susceptibility-sensitive sequences. Select 6: ≥6 CVS+ brain lesions; or the majority of lesions if fewer than 10 lesions are detected.	Typical clinical attack or progression: DIS plus Select 6 is MS 1 anatomical location plus Select 6 and either +CSF or DIT (on MRI) is MS. Incidental imaging findings of MS or presentations not specific for MS: DIS plus Select 6 is MS
Paramagnetic rim lesion (PRL)	A lesion with a discrete paramagnetic rim on susceptibility-sensitive sequence around a hyperintense lesion core. ≥1 PRL in the brain is considered positive.	Typical clinical attack or progression: 1 anatomical location plus ≥ 1 PRL and either +CSF or DIT (on MRI) is MS
CSF Kappa free light chain (kFLC)	Kappa isotype free light chain monomers suggestive of intrathecal inflammation.	Interchangeable with OCB. Typical clinical attack or progression: DIS plus +CSF is MS 1 anatomical location plus +CSF and either Select 6 or PRL (on MRI) is MS Incidental imaging findings of MS or presentations not specific for MS: DIS plus +CSF is MS
Optical coherence tomography	pRNFL and/or macular GCIPL inter-eye differences of ≥6 μm and ≥4 μm with no better explanation.	Supports optic nerve involvement as a fifth anatomical location for DIS
Visual evoked potentials	Unilateral VEP latency or asymmetric inter-ocular latencies (2.5 or 3 SD above the mean for both absolute peak P100 latency and inter-ocular latency) with no better explanation.	Supports optic nerve involvement as a fifth anatomical location for DIS
Orbital MRI	Orbital MRI with signal changes suggestive of optic neuritis, short segment, no chiasmal involvement or optic peri-neuritis.	Supports optic nerve involvement as a fifth anatomical location for DIS

GCIPL: ganglion cell-inner plexiform layer; pRNFL: retinal nerve fiber layer.

## Data Availability

No new data were created or analyzed in this study. Data sharing is not applicable to this article.

## References

[1] Dobson R., Giovannoni G. (2019). Multiple sclerosis—A review. Eur. J. Neurol..

[2] Solomon A.J., Arrambide G., Brownlee W.J., Flanagan E.P., Amato M.P., Amezcua L., Banwell B.L., Barkhof F., Corboy J.R., Correale J. (2023). Differential diagnosis of suspected multiple sclerosis: An updated consensus approach. Lancet Neurol..

[3] Spelman T., Magyari M., Piehl F., Svenningsson A., Rasmussen P.V., Kant M., Sellebjerg F., Joensen H., Hillert J., Lycke J. (2021). Treatment Escalation vs Immediate Initiation of Highly Effective Treatment for Patients with Relapsing-Remitting Multiple Sclerosis: Data from 2 Different National Strategies. JAMA Neurol..

[4] Schumacher G.A., Beebe G., Kibler R.F., Kurland L.T., Kurtzke J.F., McDowell F., Nagler B., Sibley W.A., Tourtellotte W.W., Willmon T.L. (1965). Problems of experimental trials of therapy in multiple sclerosis: Report by the panel on the evaluation of experimental trials of therapy in multiple sclerosis. Ann. N. Y. Acad. Sci..

[5] Poser C.M., Paty D.W., Scheinberg L., McDonald W.I., Davis F.A., Ebers G.C., Johnson K.P., Sibley W.A., Silberberg D.H., Tourtellotte W.W. (1983). New diagnostic criteria for multiple sclerosis: Guidelines for research protocols. Ann. Neurol..

[6] McDonald W.I., Compston A., Edan G., Goodkin D., Hartung H.P., Lublin F.D., McFarland H.F., Paty D.W., Polman C.H., Reingold S.C. (2001). Recommended diagnostic criteria for multiple sclerosis: Guidelines from the International Panel on the Diagnosis of Multiple Sclerosis. Ann. Neurol..

[7] Polman C.H., Reingold S.C., Edan G., Filippi M., Hartung H.P., Kappos L., Lublin F.D., Metz L.M., McFarland H.F., O’Connor P.W. (2005). Diagnostic criteria for multiple sclerosis: 2005 Revisions to the “McDonald Criteria”. Ann. Neurol..

[8] Polman C.H., Reingold S.C., Banwell B., Clanet M., Cohen J.A., Filippi M., Fujihara K., Havrdova E., Hutchinson M., Kappos L. (2011). Diagnostic criteria for multiple sclerosis: 2010 Revisions to the McDonald criteria. Ann. Neurol..

[9] Thompson A.J., Banwell B.L., Barkhof F., Carroll W.M., Coetzee T., Comi G., Correale J., Fazekas F., Filippi M., Freedman M.S. (2018). Diagnosis of multiple sclerosis: 2017 revisions of the McDonald criteria. Lancet Neurol..

[10] Tintore M., Cobo-Calvo A., Carbonell P., Arrambide G., Otero-Romero S., Río J., Tur C., Comabella M., Nos C., Arévalo M.J. (2021). Effect of Changes in MS Diagnostic Criteria Over 25 Years on Time to Treatment and Prognosis in Patients with Clinically Isolated Syndrome. Neurology.

[11] Kuhlmann T., Moccia M., Coetzee T., Cohen J.A., Correale J., Graves J., Marrie R.A., Montalban X., Yong V.W., Thompson A.J. (2023). Multiple sclerosis progression: Time for a new mechanism-driven framework. Lancet Neurol..

[12] Sati P., George I.C., Shea C.D., Gaitán M.I., Reich D.S. (2012). FLAIR*: A combined MR contrast technique for visualizing white matter lesions and parenchymal veins. Radiology.

[13] Maggi P., Sati P., Nair G., Cortese I.C.M., Jacobson S., Smith B.R., Nath A., Ohayon J., van Pesch V., Perrotta G. (2020). Paramagnetic Rim Lesions are Specific to Multiple Sclerosis: An International Multicenter 3T MRI Study. Ann. Neurol..

[14] Sati P., Oh J., Constable R.T., Evangelou N., Guttmann C.R.G., Henry R.G., Klawiter E.C., Mainero C., Massacesi L., McFarland H. (2016). The central vein sign and its clinical evaluation for the diagnosis of multiple sclerosis: A consensus statement from the North American Imaging in Multiple Sclerosis Cooperative. Nat. Rev. Neurol..

[15] Frischer J.M., Bramow S., Dal-Bianco A., Lucchinetti C.F., Rauschka H., Schmidbauer M., Laursen H., Sorensen P.S., Lassmann H. (2009). The relation between inflammation and neurodegeneration in multiple sclerosis brains. Brain.

[16] Bagnato F., Sati P., Hemond C.C., Elliott C., Gauthier S.A., Harrison D.M., Mainero C., Oh J., Pitt D., Shinohara R.T. (2024). Imaging chronic active lesions in multiple sclerosis: A consensus statement. Brain.

[17] Absinta M., Sati P., Schindler M., Leibovitch E.C., Ohayon J., Wu T., Meani A., Filippi M., Jacobson S., Cortese I.C.M. (2016). Persistent 7-tesla phase rim predicts poor outcome in new multiple sclerosis patient lesions. J. Clin. Investig..

[18] Borrelli S., Martire M.S., Stölting A., Bulcke C.V., Pedrini E., Guisset F., Bugli C., Yildiz H., Pothen L., Elands S. (2024). Central Vein Sign, Cortical Lesions, and Paramagnetic Rim Lesions for the Diagnostic and Prognostic Workup of Multiple Sclerosis. Neurol. Neuroimmunol. Neuroinflamm..

[19] Toosy A.T., Mason D.F., Miller D.H. (2014). Optic neuritis. Lancet Neurol..

[20] Tintore M., Rovira À., Río J., Otero-Romero S., Arrambide G., Tur C., Comabella M., Nos C., Arévalo M.J., Negrotto L. (2015). Defining high, medium and low impact prognostic factors for developing multiple sclerosis. Brain.

[21] Bsteh G., Hegen H., Altmann P., Auer M., Berek K., Di Pauli F., Kornek B., Krajnc N., Leutmezer F., MacHer S. (2023). Diagnostic Performance of Adding the Optic Nerve Region Assessed by Optical Coherence Tomography to the Diagnostic Criteria for Multiple Sclerosis. Neurology.

[22] Vidal-Jordana A., Rovira A., Arrambide G., Otero-Romero S., Río J., Comabella M., Nos C., Castilló J., Galan I., Cabello S. (2021). Optic Nerve Topography in Multiple Sclerosis Diagnosis: The Utility of Visual Evoked Potentials. Neurology.

[23] Tewarie P., Balk L., Costello F., Green A., Martin R., Schippling S., Petzold A. (2012). The OSCAR-IB consensus criteria for retinal OCT quality assessment. PLoS ONE.

[24] Tallantyre E.C., Dixon J.E., Donaldson I., Owens T., Morgan P.S., Morris P.G., Evangelou N. (2011). Ultra-high-field imaging distinguishes MS lesions from asymptomatic white matter lesions. Neurology.

[25] Solomon A.J., Schindler M.K., Howard D.B., Watts R., Sati P., Nickerson J.P., Reich D.S. (2016). “Central vessel sign” on 3T FLAIR* MRI for the differentiation of multiple sclerosis from migraine. Ann. Clin. Transl. Neurol..

[26] Castellaro M., Tamanti A., Pisani A.I., Pizzini F.B., Crescenzo F., Calabrese M. (2020). The use of the central vein sign in the diagnosis of multiple sclerosis: A systematic review and meta-analysis. Diagnostics.

[27] Toljan K., Daboul L., Raza P., Martin M.L., Cao Q., O’Donnell C.M., Rodrigues P., Derbyshire J., Azevedo C.J., Bar-Or A. (2024). Diagnostic performance of central vein sign versus oligoclonal bands for multiple sclerosis. Mult. Scler. J..

[28] Toljan K., Amin M., Ontaneda D. (2025). Central vein sign and paramagnetic rim lesion abbreviated counting methods for diagnosis of multiple sclerosis: A systematic review and meta-analysis. Clin. Radiol..

[29] Renner B., Verter E.D., Absinta M., Daboul L., Raza P., Martin M.L., Cao Q., O’Donnell C.M., Rodrigues P.R., Ramos M. (2025). Frequency and Diagnostic Implications of Paramagnetic Rim Lesions in People Presenting for Diagnosis to a Multiple Sclerosis Clinic. Neurology.

[30] Karrenbauer V.D., Bedri S.K., Hillert J., Manouchehrinia A. (2021). Cerebrospinal fluid oligoclonal immunoglobulin gamma bands and long-term disability progression in multiple sclerosis: A retrospective cohort study. Sci. Rep..

[31] Link H., Huang Y.M. (2006). Oligoclonal bands in multiple sclerosis cerebrospinal fluid: An update on methodology and clinical usefulness. J. Neuroimmunol..

[32] Saadeh R.S., Bryant S.C., McKeon A., Weinshenker B., Murray D.L., Pittock S.J., Willrich M.A.V. (2022). CSF Kappa Free Light Chains: Cutoff Validation for Diagnosing Multiple Sclerosis. Mayo Clin. Proc..

[33] Simonsen C.S., Flemmen H.Ø., Lauritzen T., Berg-Hansen P., Moen S.M., Celius E.G. (2020). The diagnostic value of IgG index versus oligoclonal bands in cerebrospinal fluid of patients with multiple sclerosis. Mult. Scler. J. Exp. Transl. Clin..

[34] Levraut M., Laurent-Chabalier S., Ayrignac X., Bigaut K., Rival M., Squalli S., Zéphir H., Alberto T., Pekar J.D., Ciron J. (2023). Kappa Free Light Chain Biomarkers Are Efficient for the Diagnosis of Multiple Sclerosis: A Large Multicenter Cohort Study. Neurol. Neuroimmunol. Neuroinflamm.

[35] Lebrun-Frénay C., Okuda D.T., Siva A., Landes-Chateau C., Azevedo C.J., Mondot L., Carra-Dallière C., Zephir H., Louapre C., Durand-Dubief F. (2023). The radiologically isolated syndrome: Revised diagnostic criteria. Brain.

[36] Okuda D.T., Mowry E.M., Beheshtian A., Waubant E., Baranzini S.E., Goodin D.S., Hauser S.L., Pelletier D. (2009). Incidental MRI anomalies suggestive of multiple sclerosis: The radiologically isolated syndrome. Neurology.

[37] Lebrun-Frenay C., Kantarci O., Siva A., Sormani M.P., Pelletier D., Okuda D.T., Azevedo C., Amato M.P., Bensa C., Berger E. (2020). Radiologically Isolated Syndrome: 10-Year Risk Estimate of a Clinical Event. Ann. Neurol..

[38] Lebrun-Frénay C., Rollot F., Mondot L., Zephir H., Louapre C., Le Page E., Durand-Dubief F., Labauge P., Bensa C., Thouvenot E. (2021). Risk Factors and Time to Clinical Symptoms of Multiple Sclerosis Among Patients with Radiologically Isolated Syndrome. JAMA Netw. Open.

[39] Matute-Blanch C., Villar L.M., Álvarez-Cermeño J.C., Rejdak K., Evdoshenko E., Makshakov G., Nazarov V., Lapin S., Midaglia L., Vidal-Jordana A. (2018). Neurofilament light chain and oligoclonal bands are prognostic biomarkers in radiologically isolated syndrome. Brain.

[40] Landes-Chateau C., Levraut M., Okuda D.T., Themelin A., Cohen M., Kantarci O.H., Siva A., Pelletier D., Mondot L., Lebrun-Frenay C. (2024). The diagnostic value of the central vein sign in radiologically isolated syndrome. Ann. Clin. Transl. Neurol..

[41] Suthiphosuwan S., Sati P., Guenette M., Montalban X., Reich D.S., Bharatha A., Oh J. (2019). The central vein sign in radiologically isolated syndrome. Am. J. Neuroradiol..

[42] Lebrun-Frénay C., Siva A., Sormani M.P., Landes-Chateau C., Mondot L., Bovis F., Vermersch P., Papeix C., Thouvenot E., Labauge P. (2023). Teriflunomide and Time to Clinical Multiple Sclerosis in Patients with Radiologically Isolated Syndrome: The TERIS Randomized Clinical Trial. JAMA Neurol..

[43] Okuda D.T., Kantarci O., Lebrun-Frénay C., Sormani M.P., Azevedo C.J., Bovis F., Hua L.H., Amezcua L., Mowry E.M., Hotermans C. (2023). Dimethyl Fumarate Delays Multiple Sclerosis in Radiologically Isolated Syndrome. Ann. Neurol..

[44] Tornatore C., Phillips J.T., Khan O., Miller A.E., Barnes C.J. (2012). Practice patterns of US neurologists in patients with CIS, RRMS, or RIS: A consensus study. Neurol. Clin. Pr..

[45] Solomon A.J., Naismith R.T., Cross A.H. (2019). Misdiagnosis of multiple sclerosis: Impact of the 2017 McDonald criteria on clinical practice. Neurology.

[46] Solomon A.J., Pettigrew R., Naismith R.T., Chahin S., Krieger S., Weinshenker B. (2021). Challenges in multiple sclerosis diagnosis: Misunderstanding and misapplication of the McDonald criteria. Mult. Scler. J..

[47] Kilsdonk I.D., Wattjes M.P., Lopez-Soriano A., Kuijer J.P.A., De Jong M.C., De Graaf W.L., Conijn M.M.A., Polman C.H., Luijten P.R., Geurts J.J.G. (2014). Improved differentiation between MS and vascular brain lesions using FLAIR* at 7 Tesla. Eur. Radiol..

[48] Maggi P., Absinta M., Grammatico M., Vuolo L., Emmi G., Carlucci G., Spagni G., Barilaro A., Repice A.M., Emmi L. (2018). Central vein sign differentiates Multiple Sclerosis from central nervous system inflammatory vasculopathies. Ann. Neurol..

[49] Allen C.M., Clarke M.A., Pai H.V., Cauchi M., Hawken J., Htet Z.M., Allen-Philbey K., Mohamed B., Fitzsimmons D., Nair R.D. (2025). Comparison of the Diagnostic Performance of the Central Vein Sign and CSF Oligoclonal Bands Supporting the Diagnosis of Multiple Sclerosis. Neurol. Open Access.

[50] Husseini L., Geladaris A., Weber M.S. (2024). Toward identifying key mechanisms of progression in multiple sclerosis. Trends Neurosci..

[51] Montalban X., Sastre-Garriga J., Filippi M., Khaleeli Z., Téllez N., Vellinga M., Tur C., Brochet B., Barkhof F., Rovaris M. (2009). Primary progressive multiple sclerosis diagnostic criteria: A reappraisal. Mult. Scler..

[52] Brownlee W.J., Vidal-Jordana A., Shatila M., Strijbis E., Schoof L., Killestein J., Barkhof F., Bollo L., Rovira A., Sastre-Garriga J. (2025). Towards a Unified Set of Diagnostic Criteria for Multiple Sclerosis. Ann. Neurol..

[53] Ruggieri S., Petracca M., Miller A., Krieger S., Ghassemi R., Bencosme Y., Riley C., Howard J., Lublin F., Inglese M. (2015). Association of deep gray matter damage with cortical and spinal cord degeneration in primary progressive multiple sclerosis. JAMA Neurol..

[54] Harrison D.M., Sati P., Klawiter E.C., Narayanan S., Bagnato F., Beck E.S., Barker P., Calvi A., Cagol A., Donadieu M. (2024). The use of 7T MRI in multiple sclerosis: Review and consensus statement from the North American Imaging in Multiple Sclerosis Cooperative. Brain Commun..

[55] Treaba C.A., Granberg T.E., Sormani M.P., Herranz E., Ouellette R.A., Louapre C., Sloane J.A., Kinkel R.P., Mainero C. (2019). Longitudinal characterization of cortical lesion development and evolution in multiple sclerosis with 7.0-T MRI. Radiology.

[56] Arnold T.C., Tu D., Okar S.V., Nair G., By S., Kawatra K.D., Robert-Fitzgerald T.E., Desiderio L.M., Schindler M.K., Shinohara R.T. (2022). Sensitivity of portable low-field magnetic resonance imaging for multiple sclerosis lesions. Neuroimage Clin..

[57] Kuhle J., Kropshofer H., Haering D.A., Kundu U., Meinert R., Barro C., Dahlke F., Tomic D., Leppert D., Kappos L. (2019). Blood neurofilament light chain as a biomarker of MS disease activity and treatment response. Neurology.

[58] Leppert D., Kropshofer H., Häring D.A., Dahlke F., Patil A., Meinert R., Tomic D., Kappos L., Kuhle J. (2022). Blood Neurofilament Light in Progressive Multiple Sclerosis: Post Hoc Analysis of 2 Randomized Controlled Trials. Neurology.

[59] Cross A.H., Gelfand J.M., Thebault S., Bennett J.L., Von Büdingen H.C., Cameron B., Carruthers R., Edwards K., Fallis R., Gerstein R. (2024). Emerging Cerebrospinal Fluid Biomarkers of Disease Activity and Progression in Multiple Sclerosis. JAMA Neurol..

[60] Amin M., Martínez-Heras E., Ontaneda D., Prados Carrasco F. (2024). Artificial Intelligence and Multiple Sclerosis. Curr. Neurol. Neurosci. Rep..

[61] Collorone S., Coll L., Lorenzi M., Lladó X., Sastre-Garriga J., Tintoré M., Montalban X., Rovira À., Pareto D., Tur C. (2024). Artificial intelligence applied to MRI data to tackle key challenges in multiple sclerosis. Mult. Scler. J..

[62] Diaz-Hurtado M., Martínez-Heras E., Solana E., Casas-Roma J., Llufriu S., Kanber B., Prados F. (2022). Recent advances in the longitudinal segmentation of multiple sclerosis lesions on magnetic resonance imaging: A review. Neuroradiology.

[63] Maggi P., Fartaria M.J., Jorge J., La Rosa F., Absinta M., Sati P., Meuli R., Pasquier R.D., Reich D.S., Cuadra M.B. (2020). CVSnet: A machine learning approach for automated central vein sign assessment in multiple sclerosis. NMR Biomed..

